# Mdfi Promotes C2C12 Cell Differentiation and Positively Modulates Fast-to-Slow-Twitch Muscle Fiber Transformation

**DOI:** 10.3389/fcell.2021.605875

**Published:** 2021-01-22

**Authors:** Bo Huang, Yiren Jiao, Yifan Zhu, Zuocheng Ning, Zijian Ye, Qing X. Li, Chingyuan Hu, Chong Wang

**Affiliations:** ^1^National Engineering Research Center for Breeding Swine Industry, Guangdong Provincial Key Lab of Agro-Animal Genomics and Molecular Breeding, Guangdong Laboratory for Lingnan Modern Agriculture, College of Animal Science, South China Agricultural University, Guangzhou, China; ^2^Department of Molecular Biosciences and Bioengineering, University of Hawai’i at Mānoa, Honolulu, HI, United States; ^3^Department of Human Nutrition, Food and Animal Sciences, University of Hawai’i at Mānoa, Honolulu, HI, United States

**Keywords:** Mdfi, C2C12 cells, CRISPR/Cas9 system, RNA-seq, differentiation, muscle fiber type transformation

## Abstract

Muscle development requires myoblast differentiation and muscle fiber formation. Myod family inhibitor (Mdfi) inhibits myogenic regulatory factors in NIH3T3 cells, but how Mdfi regulates myoblast myogenic development is still unclear. In the present study, we constructed an Mdfi-overexpression (Mdfi-OE) C2C12 cell line by the CRISPR/Cas9 system and performed RNA-seq on Mdfi-OE and wild-type (WT) C2C12 cells. The RNA-seq results showed that the calcium signaling pathway was the most significant. We also established the regulatory networks of Mdfi-OE on C2C12 cell differentiation and muscle fiber type transformation and identified hub genes. Further, both RNA-seq and experimental verification demonstrated that Mdfi promoted C2C12 cell differentiation by upregulating the expression of Myod, Myog, and Myosin. We also found that the positive regulation of Mdfi on fast-to-slow-twitch muscle fiber transformation is mediated by *Myod*, *Camk2b*, and its downstream genes, such as *Pgc1a*, *Pdk4*, *Cs*, *Cox4*, *Acadm*, *Acox1*, *Cycs*, and *Atp5a1*. In conclusion, our results demonstrated that Mdfi promotes C2C12 cell differentiation and positively modulates fast-to-slow-twitch muscle fiber transformation. These findings further our understanding of the regulatory mechanisms of Mdfi in myogenic development and muscle fiber type transformation. Our results suggest potential therapeutic targets for muscle- and metabolic-related diseases.

## Introduction

Skeletal muscle accounts for about 45% of the human body weight (Turner and Badylak, 2012). Skeletal muscle development plays a crucial role in maintaining the muscle loss caused by disease, injury, and aging. Thus, it is of considerable significance to understand the mechanism of muscle myogenic development for preventing adverse effects on human health.

The skeletal muscle tissue maintains homeostasis through skeletal muscle satellites self-renewal and differentiation when suffered from pathological change or injury (Fry et al., 2015). When stimulated by injury or growth signals, skeletal muscle satellite cells are activated and proliferate to form myoblasts (Abou-Khalil et al., 2009). Subsequently, myoblasts exit the cell cycle, and the myogenic regulator factor (MRF) family, including myogenic factor 5 (Myf5), myogenic differentiation 1 (MyoD), myogenin (Myog), and myogenic factor 6 (Myf6), gradually expresses (Hernandez-Hernandez et al., 2017b). Myf5 is first expressed when satellite cells are activated. Previous studies have found that Myf5 transforms many non-muscle cells into muscle cells (Delfini et al., 2000). MyoD began to express at the proliferation phase of myoblasts and mainly maintains its early differentiation (Zammit et al., 2004). Myog is not expressed in the proliferation phase of myoblasts, but its expression is significantly upregulated when the cells entered the differentiation phase (Bentzinger et al., 2012). Myf6 mainly regulates the terminal differentiation of myoblasts and is highly expressed in mature muscle fibers (Bentzinger et al., 2012). As a result, these myoblasts undergo differentiation to either repair damaged muscle fibers or fuse into multi-nuclear myotubes to form new myofibers (Mashinchian et al., 2018). Myogenic differentiation is, therefore, an essential process in muscle development which determines myoblast fate, muscle formation, and regeneration. Elucidating the mechanism of muscle cell differentiation is critical for understanding skeletal muscle development.

Skeletal muscles are composed of multi-nucleated cells that have numerous myofibers (Bassel-Duby and Olson, 2006). Based on myosin heavy chains (MyHC), mature mammalian skeletal muscle fibers can be classified as type I (MyHC I), type IIa (MyHC IIa), type IIx (MyHC IIx), and type IIb (MyHC IIb) (Schiaffino and Reggiani, 2011). Slow-twitch muscle is mainly composed of type I and type IIa muscle fibers, which are rich in myoglobin and mitochondria, have a strong fatigue resistance. Type IIb muscle fibers mainly exist in fast-twitch muscles, displaying low myoglobin and mitochondrial content, and have weak resistance to fatigue resistance (Chen et al., 2018). Skeletal muscle fiber type transformation is directly correlated with some human muscular and metabolic diseases. For example, muscle atrophy leads to an increase in the proportion of slow-twitch muscle fibers and a decrease in the proportion of fast-twitch muscle fibers (Wang and Pessin, 2013). In the skeletal muscles of patients with type 2 diabetes, the proportion of slow oxidative fibers is decreased, which leads to a decrease in oxidative enzyme activity (Oberbach et al., 2006). Chronic liver disease (CLD) led to the transformation of muscle fibers from type IIb to type I (Aguirre et al., 2020). Chronic obstructive pulmonary disease (COPD) significantly increases the expression of tumor necrosis factor (TNF)-like weak inducer of apoptosis (TWEAK), then increases the proportion of type 1 muscle fibers and decreases the proportion of type 2 muscle fibers (Lu et al., 2017). Therefore, elucidating the mechanism of muscle fiber type transformation is essential for preventing adverse health effects.

Myod family inhibitor (Mdfi, also known as I-mfa) is a class of novel myogenic repressor. The yeast two-hybrid experiments showed that Mdfi inhibits the Myod family’s transactivation activity and leads to repressing the myogenic differentiation of NIH-3T3 cells (Chen et al., 1996). However, Huang et al. (2019) found that in chicken primary myoblasts overexpression of the MyoD family inhibitor domain-containing protein (MDFIC) promotes myotubes’ formation, while it shows opposite results after the knockdown of MDFIC (Huang et al., 2019). MDFIC shares a high degree homology C-terminal domain with Mdfi (Thebault and Mesnard, 2001). In addition, miR-27b inhibits Mdfi to regulate the development of pig muscle satellite cells (PSCs) *in vitro* (Hou et al., 2018). Conflicting reports on the function of Mdfi exist. We, therefore, explored the regulatory mechanisms of Mdfi in muscle development in the present study.

In this study, we constructed a Mdfi-overexpressing C2C12 cell line by the CRISPR/Cas9 system and performed RNA-seq on Mdfi overexpression (Mdfi-OE) and wild-type (WT) C2C12 cells. Real-time quantitative polymerase chain reaction (qPCR), Western blot, immunofluorescence, and RNA-seq analyses demonstrated that Mdfi promotes C2C12 cell differentiation by upregulating the expression of Myod and myogenin and positively modulates muscle fiber transformation, and successfully established the regulatory network. This study furthers our understanding of the regulatory mechanisms of Mdfi in myogenic differentiation and muscle fiber type transformation. Our results help develop new strategies for treating muscle- and metabolic-related diseases.

## Materials and Methods

### C2C12 Cell Culture, Transfection, and Differentiation

The C2C12 cell line (ATCC^®^, CRL-1772^TM^) used in this study was purchased from American Type Culture Collection (ATCC, VA, United States). The pX330-U6-Chimeric_BB-CBh-hSpCas9 (pX330, #42230) was purchased from Addgene (Cambridge, MA, United States). C2C12 cells were cultured in Dulbecco’s Modified Eagle Medium (DMEM)/High Glucose (Catalog No. SH30243.01, Hyclone, GE Healthcare Bio-Sciences, Pittsburgh, PA, United States) with 10% Fetal Bovine Serum (FBS) (Catalog No. FBS10099-141, Gibco, Grand Island, NY, United States). C2C12 cells were seeded in 6-well plates (2 × 10^5^ cells per well). When plates reached 80–90% confluence, the cells were cultured by myogenic differentiation induction medium. C2C12 cells transfected with pX330, pX330-sgRNA plasmid, or co-transfected with pEGFP by Lipofectamine 2000 (Invitrogen, Carlsbad, CA, United States), according to the manufacturer’s instructions. The medium was replaced with fresh growth medium 6 h later.

### Construction of a Mdfi-Overexpressing Cell Line by CRISPR/Cas9

We constructed a Mdfi-overexpressing C2C12 cell line by inserting a Mdfi transgene cassette into the genome ROSA26 locus using the CRISPR/Cas9 system. The Genome-CRISPR^TM^ mouse ROSA26 safe harbor gene knock-in kit was purchased from GeneCopoeia Inc (Catalog No. SH-ROS-K200, GeneCopoeia Inc., Rockville, MD, United States). We transfected the MCP-ROSA26-CG01 vector into C2C12 cells with DC-DON-SH02, Mdfi donor, and DC-RFP-SH02. After transfection for 24 h, puromycin (2 μg/mL) was used to screen Mdfi-overexpressing monoclonal cells. After puromycin screening for 72 h, we obtained Mdfi-overexpressing monoclonal cells using limiting dilution assay.

### RNA Extraction and qPCR Analysis

The methods used for the RNA extraction and PCR analysis have been described previously (Hou et al., 2017). Briefly, total RNAs were extracted from C2C12 cells using TRIzol reagent (Invitrogen) according to the manufacturer’s instructions. After DNase I (Takara Bio Inc., Japan) digestion, total RNAs (500 ng) were reverse transcribed to cDNA using PrimeScript^TM^ RT Master Mix (TaKaRa, Otsu, Shiga, Japan). SYBR Green Real-time PCR Master Mix reagents (Toyobo Co., Ltd., Osaka, Japan) were used for qPCR. The PCR reactions were carried out on a CFX96^TM^ Optical Reaction Module (Bio-Rad, Hercules, CA, United States). The relative expression of mRNAs was normalized with β-actin levels using the ΔΔCt method. Primers used for qPCR are shown in Supplementary Table 1.

### Immunofluorescent Assay

Wild-type and Mdfi-OE C2C12 cells were seeded in the 48-well at a density of 5 × 10^4/^mL and maintained in the growth medium. When cells reached 90% confluence, we changed the growth medium to the differentiation medium (2% house serum) for induction differentiation. At differentiation for 1, 3, 5, and 7 days, we removed the old medium and washed the C2C12 cells three times by PBS. The C2C12 cells were fixed for 20 minutes by 80% acetone, permeabilized for 10 min by 0.5% Triton^TM^ X-100 (Sigma-Aldrich, St. Louis, MO, United States). We used the BCA protein assay kit (Dingguo, China) to block for 1 h, followed by incubating the C2C12 cells with the primary myosin antibody for 1 h. We incubated the C2C12 cells with secondary antibody for 30 min. Finally, we incubated the C2C12 cells with DAPI. The myosin-positive C2C12 cells were observed and recorded using a Nikon TE2000-U inverted microscope (Nikon Instruments, Tokyo, Japan). More than six fields of view were captured in each cell well. The percentage of myosin-positive C2C12 cells, calculated as the number of nuclei present in multi-nucleic myotubes (myosin-positive and containing at least three nuclei) in comparison with the total number of nuclei. The percentage of MyHC-positive C2C12 cells was calculated as the number of nuclei present in multi-nucleic myotubes (MyHC I, MyHC IIa, and MyHC IIb -positive, and containing at least three nuclei) relative the total number of nuclei. Data were counted with Image J software (National Institutes of Health, Bethesda, MD, United States). They were analyzed with the GraphPad Prism (GraphPad Software, La Jolla, CA, United States). The data were expressed as the mean ± standard error of the mean (SEM) with SPSS software (SPSS, Inc., Chicago, IL, United States).

### Immunoprecipitation

C2C12 cells were washed twice with precooled PBS, PBS was finally drained, and a 1mL precooled modified RIPA Buffer was added to the petri dish. The cell suspension was transferred to a 1.5 mL centrifuge tube at 4°C for 15 min. After standing, centrifuge at 14,000*g* for 15 min, and transfer the supernatant to a new centrifuge tube. The protein A agarose beads was washed twice with PBS and then prepared into 50% concentration with PBS. About 100 μL 50% protein A agarose beads was added to every 1 mL total protein and incubated at 4°C for 10 min. The supernatant was centrifuged at 4°C, 14,000 rpm for 15 min, and the supernatant was transferred to a new centrifuge tube to remove the protein A beads. The total protein was diluted at least 1:10 times, diluted to about 1 μg/μL with PBS, and incubated overnight at 4°C. An aliquot of 100 μL protein A agarose beads was then added into the centrifuge tube to capture the antigen-antibody complex. The mixture was shaken for 24 h at 4°C. After centrifugation at 14,000 rpm for 5 s, the agarose beads antigen-antibody complex was collected and the supernatant was removed. The agarose beads were washed with precooled NP-40 lysate three times. After washing, an aliquot of 60 mL of 2 × SDS-PAGE buffer was added in the centrifuge tube, mixed gently, add boiled with boiling water for 5 min. After centrifugation, the supernatant was transferred to a new centrifuge tube for subsequent electrophoresis and the remaining agarose beads were collected.

### Luciferase Reporter Assay (Promoter Activity Detection)

The genomic DNA of C2C12 cells was isolated for the PCR amplification template. The promoter of the *Camk2b* gene was amplified by PCR using PrimerSTAR^®^ (TaKaRa, Dalian, Liaoning, China). Then, we cloned the promoter of the *Camk2b* gene into the eukaryotic expression vector pGL3-Basic (named as pGL3-Basic-Camk2b). Similarly, we amplified the full length of the *Myod* gene and cloned it into the pcDNA3.1 plasmid (pcDNA3.1-Myod). According to Promega’s dual luciferase reporter assay kit (Promega, Madison, WI, United States), we transfected the pGL3-Basic, pGL3-Basic-Camk2b and pcDNA3.1, pGL3-Basic-Camk2b and pcDNA3.1-Myod into C2C12 cell by Lipofectamine^TM^ 3000 Transfection Reagent (Thermo Fisher Scientific, MA, United States). At last, we identified the double-luciferase activity by BioTek Synergy 2 multifunctional microplate reader (BioTek, Winooski, VT, United States). The ratio of the expression of firefly luciferase to renilla luciferase was the promoter activity. The PCR primer pairs were listed in Supplementary Table 1.

### Chromatin Immunofluorescent Assay

The chromatin immunoprecipitation (ChIP)-IT^®^ Express Magnetic ChIP kit & sonication shearing kit (Catalog number 53008) was purchased from Active Motif (Carlsbad, CA, United States). For the ChIP assay, the DNA was immunoprecipitated with the Myod or Camk2b antibody, and ChIP analysis was performed according to the manufacturer’s protocol. DNA samples prior to immunoprecipitation were used as a template for input control. Primers used for ChIP assay are shown in Supplementary Table 1. The antibodies used for ChIP assay are shown in Supplementary Table 1.

### Western Blot Assay

Methods used for Western blot assay have been described previously (Hou et al., 2017). C2C12 cells lysed in RIPA buffer containing 1 μM phenylmethanesulfonyl fluoride (PMSF). About 30 μg protein lysates were separated using SDS-PAGE and then electroblotted onto polyvinylidene fluoride membranes (Bio-Rad). The membranes were blocked with 6% skim milk buffer for 2 h at room temperature and incubated with different diluted antibodies at 4°C overnight. Finally, the polyvinylidene fluoride membranes were incubated with horseradish peroxidase-conjugated secondary antibodies at room temperature for 1 h. The antibodies used in this study are listed in Supplementary Table 2. The band intensities were quantified with Image J and normalized to those of β-actin. Data were analyzed using the GraphPad Prism and were expressed as change in fold relative to the control.

### Mitochondrial DNA Copy Number

Total cellular DNA was extracted from WT and Mdfi-OE C2C12 cells with DNAzol reagent (Invitrogen). Mitochondrial 16S ribosomal RNA (mito-16sRNA) was used as the internal reference gene of mitochondrial DNA, and the hexokinase gene (Mito-HEXO) was used as the internal reference gene of nuclear DNA. The expression level of mito-16sRNA relative to Mito-HEXO was quantified, and the expression of mito-16sRNA reflected the copy number of mitochondrial DNA.

### RNA-seq Analysis

Wild-type and Mdfi-OE C2C12 cell samples were sequenced using 150 bp paired-end mRNA sequencing methods based on the Illumina HiSeq platform (Caporaso et al., 2012). We used FastQC software to check Pass Filter Data quality. Cutadapt (version 1.9.1), a second-generation software of sequencing data quality statistics, was used to remove the adapter and low-quality sequences from the raw data and obtain clean data (clean reads). After quality control, clean reads were mapped to the mouse reference genome (*Mus musculus* 10.fa) using Hisat2 with one mismatch tolerance. We acquired expression levels based on the read counts using Subread (Liao et al., 2019). Differential expression analysis was performed on DESeq2 software. *p* < 0.05 and | log2FoldChange| > 1 was set as the cutoff criteria for differentially expressed analysis. Kyoto Encyclopedia of Genes and Genomes (KEGG) enrichment analyses of genes were conducted using KOBAS 3.0 (kobas.cbi.pku.edu.cn) (Xie et al., 2011). The KEGG database analysis was used to analyze the enriched pathways for the differentially expressed genes (DEGs). The Benjamini-Hochberg procedure was applied to control the false discovery rate (*p* < 0.05). String database v10.5 (string-db.org) was used to establish the protein–protein interactions (PPI) of DEGs between the Mdfi-OE and WT groups with the criterion of medium confidence (0.700) (Szklarczyk et al., 2019). Moreover, we applied Cytoscape 3.6 (Shannon et al., 2003) to visualize the network and highlight the most representative gene.

### Statistical Analysis

All data are expressed as the mean ± SEM. There were three replicates in each group. The assumptions of normality of data and homogeneity of variances between the groups were analyzed by SPSS. The dual-luciferase reporter system data were analyzed by one-way ANOVA (SPSS 18.0, Chicago, IL, United States). Significant differences between the control and the treatment groups were determined using the Student’s *t*-test. We considered *p* < 0.05 to be statistically significant. ^∗^ is *p* < 0.05 and ^∗∗^ is *p* < 0.01.

## Results

### The Expression Profile of Mdfi During C2C12 Cell Differentiation

**FIGURE 1:**
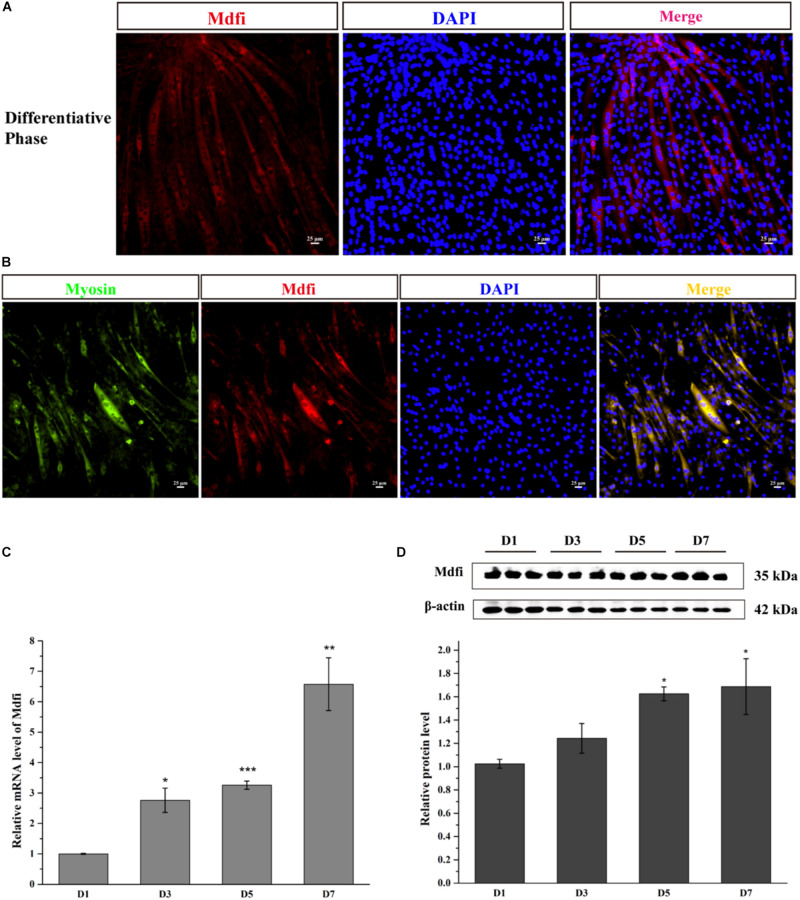
Mdfi-expression profile during C2C12 cell development. **(A)** Mdfi immunofluorescence of C2C12 cells in differentiative phase. **(B)** Mdfi and myosin co-immunofluorescence of C2C12 cells in differentiative phase. Myosin: green, a molecular marker of myogenesis; Mdfi: red; DAPI: blue, cell nuclei; Merge: C2C12 cells fused into multi-nucleic myotubes. **(C)** qPCR identified Mdfi mRNA level on days 1, 3, 5, and 7 after differentiation induction. **(D)** Western blot identified Mdfi protein level on days 1, 3, 5, and 7 after differentiation induction. *p < 0.05; **p < 0.01; ***p < 0.001. The results are presented as Mean ± SEM of three replicates for each group. Magnification 200×. The scale bar on the photomicrographs represents 25 μm.

Myod family inhibitor is expressed mainly in the cytoplasm of C2C12 cells, which have been fused into myotube (Figure 1A). The Mdfi and myosin co-immunofluorescent assay results showed that Mdfi expressed mainly in the cytoplasm of myosin positive C2C12 cells (Figure 1B). Meanwhile, we identified the mRNA and protein level of Mdfi in the differentiation phase of C2C12 cells by qPCR and Western blot. The qPCR results showed that the mRNA level of *Mdfi* was increased at day 3 (*p* < 0.05), day 5 (*p* < 0.001), and day 7 (*p* < 0.01) compared to day 1 (Figure 1C). The Western blot results are consistent with the qPCR results. The protein level of Mdfi was higher on day 5 (*p* < 0.05) and day 7 (*p* < 0.05) than day 1 in the differentiation phase (Figure 1D). Although the protein level of Mdfi was not increased significantly at day 3 (*p* > 0.05) compared to day 1, there was still an increasing trend. These results indicated that the expression of Mdfi was significantly increased during myoblast differentiation.

### Construction of a Mdfi-OE C2C12 Cell Line by CRISPR/Cas9

**FIGURE 2:**
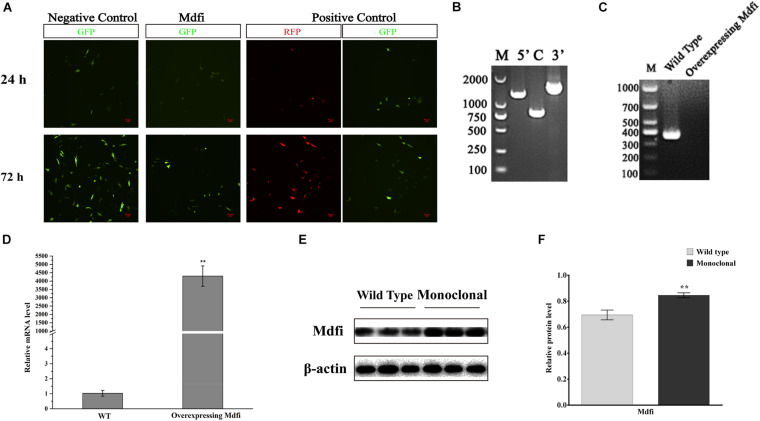
Construction of a Mdfi-overexpressing C2C12 cell line. **(A)** Inverted fluorescence microscopy was used to investigate the expression of GFP and RFP protein in the C2C12 cell after puromycin screening for 24 and 72 h after transfected with Rosa26 + Donor, Rosa26 + Donor-Mdfi, and Rosa26+ Donor-RFP, respectively. **(B)** The homologous recombination status of left and right homologous arms and the insertion of the Mdfi gene in the monoclonal clone was confirmed. **(C)** Single or double allele insertion of Mdfi in monoclonal clone was confirmed through PCR amplification. **(D)** qPCR measured the mRNA level of Mdfi. **(E)** Western blot measured the protein level of Mdfi. **(F)** The relative protein levels obtained through Western blot band gray scanning analysis. ***p < 0.001. The results are presented as Mean ± SEM of three replicates for each group.

To explore the function of Mdfi in C2C12 myogenic development, we used the CRISPR/Cas9 system to construct a stable Mdfi-overexpressing (Mdfi-OE) C2C12 cell line. Immunofluorescent assay results showed that we have successfully inserted Mdfi into C2C12 cells at ROSA26 locus, which provided candidate Mdfi-OE monoclonal cells (Figure 2A). The PCR amplification results showed that the left and right homologous arms were successfully recombined, indicating the correct integration of the Mdfi donor at the ROSA26 locus (Figure 2B). Results of PCR amplification showed that the non-insertion fragment in the Mdfi-OE monoclonal cells could not be amplified (Figure 2C). These results indicated that monoclonal C2C12 cells represent a heterozygous C2C12 Mdfi-OE cell line. The qPCR results showed that the mRNA level of *Mdfi* was significantly upregulated compared with the WT (*p* < 0.01) (Figure 2D). The Western blot results showed that the Mdfi protein level of monoclonal cells was significantly increased compared to WT (*p* < 0.01), indicating that Mdfi successfully overexpressed in monoclonal cells (Figures 2E,F). These results indicated that we have successfully constructed a Mdfi-OE C2C12 cell line to explore the function of Mdfi in C2C12 myogenic development.

### Characteristics of Differentiation and Muscle Fiber Types in C2C12 Cells With Mdfi-OE

**FIGURE 3:**
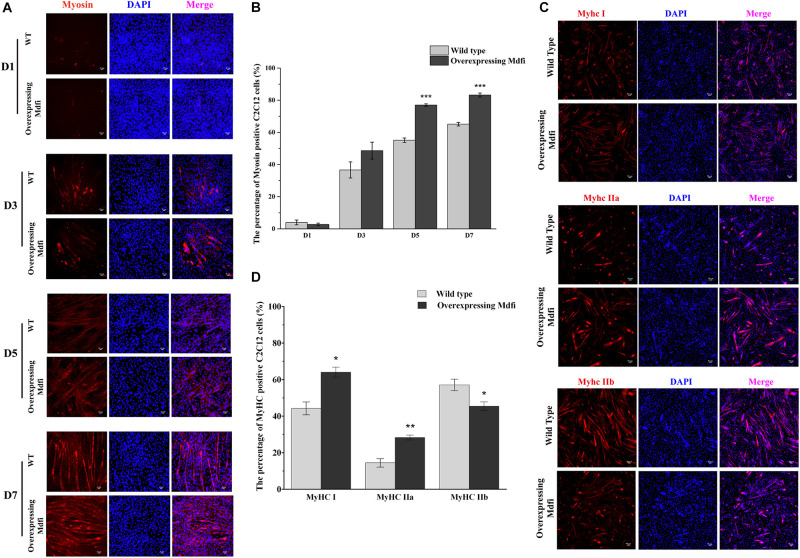
Characteristics of differentiation and muscle fiber types in C2C12 cells with Mdfi overexpression. **(A)** Representative images of differentiating wild-type and overexpressing Mdfi C2C12 cells on days 1, 3, 5, and 7 in white light or myosin immunofluorescent staining. Myosin: red, a molecular marker of myogenesis; DAPI: blue, cell nuclei; Merge: the C2C12 cells fused into primary myotubes are shown in the insets. **(B)** Quantitative diagram of the percentage of myosin-positive C2C12 cells. **(C)** MyHC I, MyHC IIa, and MyHC IIb immunofluorescence staining in WT and Mdfi-OE C2C12 cells, respectively. **(D)** Quantitative diagram of the percentage of MyHC I, MyHC IIa, and MyHC IIb-positive C2C12 cells. The results are presented as Mean ± SEM of three replicates for each group. Magnification 200×. The scale bar on the photomicrographs represents 25 μm.

To further investigate the potential role of Mdfi in the differentiation of C2C12 cells, we induced the WT and Mdfi-OE C2C12 cells in the differentiated medium at D1, 3, 5, and 7. Under white light, we observed that Mdfi-OE C2C12 cells fused into thicker myotubes than the WT group (Supplementary Figure 1). Myosin immunofluorescent staining results showed that Mdfi significantly increased the percentage of the myosin-positive cells at D5 (*p* < 0.001) and D7 (*p* < 0.001) (Figures 3A,B). In addition, we performed immunofluorescent staining on different subtypes of MyHC. The results showed that overexpression of Mdfi significantly increased the percentage of MyHC I (*p* < 0.05) and MyHC IIa (*p* < 0.01) positive C2C12 cells (Figure 3C). In contrast, overexpression of Mdfi decreased the percentage of MyHC IIb (*p* < 0.05) positive C2C12 cells (Figures 3C,D).

### RNA-seq Analysis in Mdfi-OE vs WT C2C12 Cells

**FIGURE 4:**
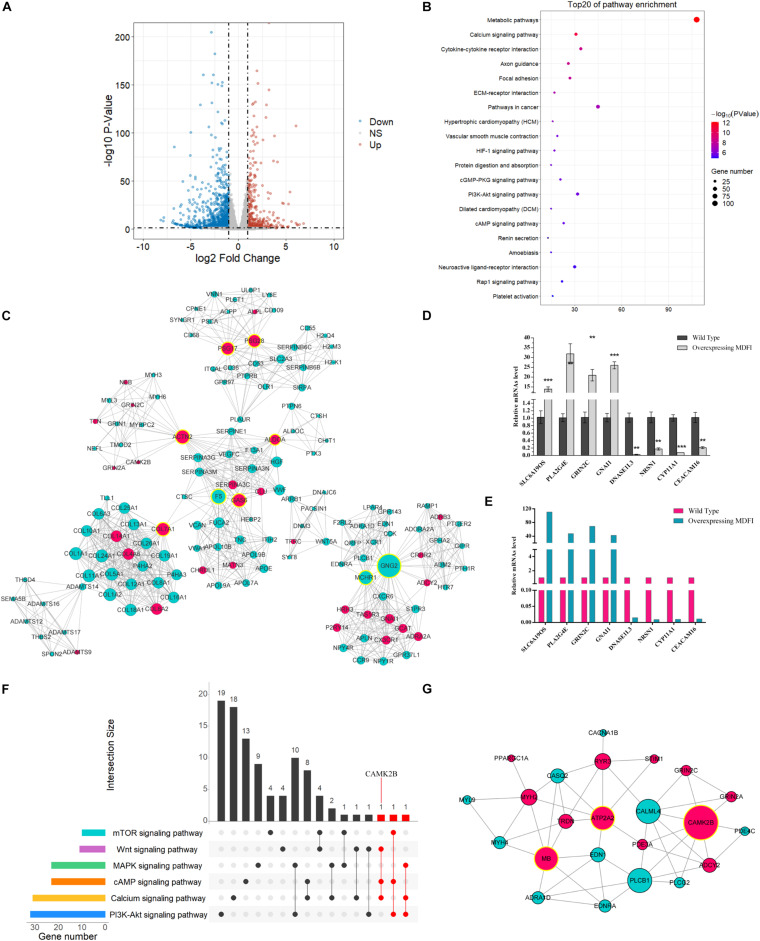
RNA-seq analysis in Mdfi-OE vs. WT C2C12 cells. **(A)** The Volcano plot displays an overview of the DEGs. The X-axis represents the log-transformed *P*-value, and the Y-axis indicates the multiple of the DEGs. The gray dots represent DEGs that are not differentially expressed, the red dots represent the upregulated DEGs, and the blue dots represent the downregulated DEGs. |Log2FoldChange| > 1 and *p* < 0.05 were set as the criteria. **(B)** The first twenty pathways of the DEGs. The X-axis represents the number of DEGs enriched in the pathway. **(C)** PPI network in Mdfi-OE vs. WT. The network displays gene interactions. Nodes represent genes, and edges represent gene interactions. The upregulated genes are shown in red, downregulated genes are shown in green, and hub genes are shown in yellow. **(D)** qPCR validated of DEGs. **(E)** Expression levels of DEGs in RNA-seq. **p < 0.01, ***p < 0.001. **(F)** The upset plot of the intersection of each pathway. The transverse bar graph at the bottom left shows the number of genes enriched in each pathway. The black points in the dot matrix at the bottom left and in the bar graph at the top indicate genes included in the pathways. The bars above represent the number of genes corresponding to each intersection. The red bars represent the number of intersection genes enriched in three or more pathways (*n* ≥ 3). **(G)** The gene network displayed the gene interactions and was generated by Cytoscape (version 3.6). Nodes represent genes, and edges represent gene interactions. The upregulated genes are shown in red, downregulated genes are shown in green, and hub genes are shown in yellow.

To further investigate the function of Mdfi in C2C12 myoblast differentiation, we performed RNA-Seq to profile genes expressed. The mRNA profiles were generated by sequencing six C2C12 cell samples (three each from Mdfi-OE and WT). Sample’s length, clean reads, Q30 quality scores, GC content, and unique mapped rate were summarized in Table 1. A total of 254 million clean reads were retrieved from the mRNA profiles. The length of the clean reads ranged from 147.69 to 148.30 nt. Q30 quality scores of the six samples were above 92%. The GC content of the samples ranged from 49 to 50%. The unique mapped rate was above 83%. Based on the criteria of *p* < 0.05 and | Log2FoldChange| > 1, we identified 1,522 DEGs between Mdfi-OE and WT groups (Supplementary Table 3). Among these DEGs, 434 genes were upregulated, and 1,088 genes were downregulated in Mdfi-OE vs. WT (Figure 4A). *Mdfi* was markedly upregulated by 64-fold. Based on the KEGG analysis, 94 pathways were significantly enriched with a *p* < 0.05 (Supplementary Table 4). The first 20 KEGG pathways were shown in Figure 4B. The “calcium signaling pathway” was the most enriched. Furthermore, we established a PPI network composed of 1,522 DEGs, 287 nodes, and 1,024 edges (Figure 4C). High degreed-nodes in the regulatory network are also known as hub genes. We identified nine hub genes in the gene network, including six upregulated genes and three downregulated genes. These genes all play the key roles in the differentiation of the C2C12 cells with overexpressing Mdfi, which warrants further investigations. To validate the results of RNA-seq, we used qPCR to validate changes in expression levels of C2C12 cells overexpressed with Mdfi with induced differentiation. The qPCR results for all the DEGs were consistent with the results of the RNA-seq data (*p* < 0.01) (Figures 4D,E).

**TABLE 1:** Overview of mRNA sequencing data.

Samples	ID	Length	Clean reads	Q30 (%)	GC (%)	Unique mapped reads (%)
Wild-Type 1	DC1	148.29	42843674	92.47	50.40	50.40
Wild-Type 2	DC2	148.29	41911008	92.10	49.85	49.85
Wild-Type 3	DC3	148.30	47325970	92.39	49.69	49.69
Mdfi-OE 1	DE1	147.85	39635016	92.06	49.15	49.15
Mdfi-OE 2	DE2	147.69	40636938	92.11	49.11	49.11
Mdfi-OE 3	DE3	148.07	41973956	92.71	49.52	49.52

*Samples: Wild-Type 1/2/3 represent normal C2C12 cells. Mdfi-OE 1/2/3 represent overexpression of Mdfi in C2C12 cells. ID represents sample serial numbers. Length represents the length of sequencing reads. Clean reads were done after removing impurity reads for raw reads. Q30% represents the percentage of bases with mass values greater than or equal to 30. GC Content is the G and C base content. Unique mapped reads are the percentage of each sample aligned to the mouse reference genome.*

As expected, our RNA-seq analysis results showed that the IIa muscle fiber marker gene, myosin heavy chain 2 (*Myh2*) (about 1.33-fold), and myoglobin (*Mb*) (about 1.60-fold) were upregulated. In contrast, the IIb muscle fiber marker gene, myosin heavy chain 4 (*Myh4*) (about 2.46-fold), was downregulated. Furthermore, we identified six pathways involved in muscle fiber transformation in our RNA-seq results, such as “calcium signaling pathway,” “PI3K-Akt signaling pathway”, and “mTOR signaling pathway” (Table 2). In addition, we analyzed the intersection of genes in these pathways. The more pathways the gene is involved in, the more likely it is engaged in Mdfi regulating the transformation of muscle fiber types. As shown in the gene Venn diagram, calcium/calmodulin-dependent protein kinase II beta (*Camk2b*) was enriched in three pathways (Figure 4F). We then selected genes enriched in the above pathways to establish a regulatory network (Figure 4G); we found that most of the genes, especially *Camk2b*, ATPase sarcoplasmic/endoplasmic reticulum calcium ion transporting 2 (*Atp2a2*), and myoglobin (*Mb*), were related to calcium-induced muscle fiber type transformation. Particularly, *Camk2b* was the most degree hub gene in the gene network.

**TABLE 2:** The significantly enriched pathways related to muscle fiber type transformation.

Term	Count	*p*-Value
Calcium signaling pathway	31	1.41E-11
PI3K-Akt signaling pathway	32	1.99E-06
cAMP signaling pathway	23	3.26E-06
mTOR signaling pathway	10	0.043912
Wnt signaling pathway	11	0.026485
MAPK signaling pathway	23	0.000322

### Mdfi-OE Promoted C2C12 Cells Myogenic Differentiation

**FIGURE 5:**
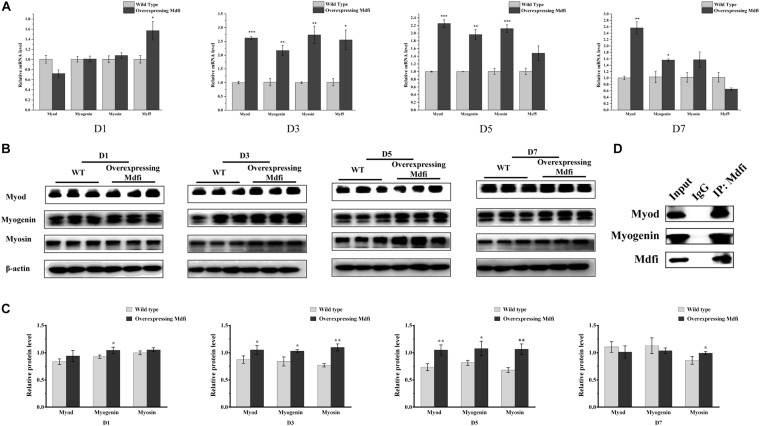
Mdfi promotes the differentiation of C2C12 cells. **(A)** qPCR detection of myogenic marker genes on days 1, 3, 5, and 7 in both WT and Mdfi-OE C2C12 cells. **(B)** Western blot identified the protein of myogenic marker genes on days 1, 3, 5, and 7 after differentiation induction in both WT and Mdfi-OE C2C12 cells. **(C)** The relative protein levels were obtained through Western blot band gray scanning analysis. **(D)** Co-IP identified the interaction of Mdfi with Myod and Myog, respectively. *p < 0.05; **p < 0.01; ***p < 0.001. The results are presented as Mean ± SEM of three replicates for each group.

To further validate the regulatory mechanism of Mdfi on C2C12 cell differentiation, we performed additional experiments. The qPCR results showed that overexpression of Mdfi significantly increased the mRNA level of *Myod* (*p* > 0.001), *Myog* (*p* > 0.01), and *Myosin* (*p* > 0.001) mainly at D3 and D5 (Figure 5A). There was no significant difference in mRNA levels of Myod, *Myog*, and *Myosin* between Mdfi-OE and WT at D1 (Figure 5A). The Western blot results showed that Mdfi significantly increased the Myod protein level in D3 and D5, Myog protein level in D1, D3, and D5, and Myosin protein level in D3, D5, and D7 (Figures 5B,C). Although the Myod and Myosin protein level of Mdfi-OE was not increased significantly at D1 compared to WT, there was still an increasing trend (Figures 5B,C). There was no significant difference in protein levels of Myod and Myog between Mdfi-OE and WT at D7 (Figures 5B,C). Furthermore, the Co-IP results showed that Mdfi interacted with both Myod and Myog (Figure 5D). These results indicated that Mdfi promotes C2C12 myogenic differentiation by upregulating the expression of Myod, Myog, and Myosin.

### Mdfi Promotes the Muscle Fiber Type From the Fast-Twitch Muscle Fiber to the Slow-Twitch Muscle Fiber

**FIGURE 6:**
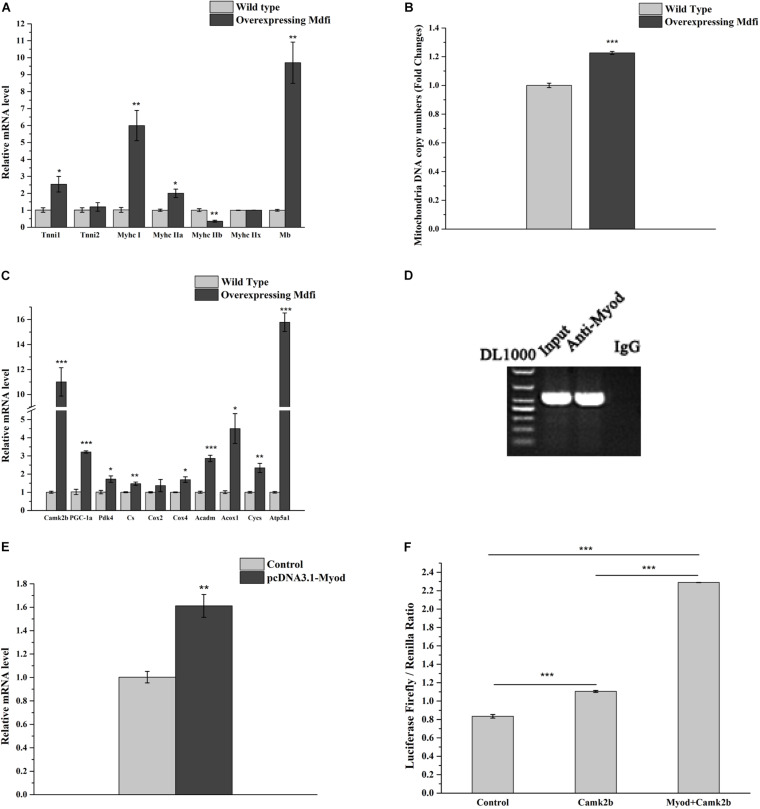
Mdfi positively regulated the transformation of muscle fiber types from fast-twitch muscle to slow-twitch muscle in C2C12 cells. **(A)** qPCR identified the mRNA level of marker genes of different muscle fiber types in WT and Mdfi-OE C2C12 cells, respectively. **(B)** qPCR identified the relative copy number of mitochondrial DNA in WT and Mdfi-OE C2C12 cells, respectively. **(C)** qPCR identified the mRNA levels of energy metabolism-related genes in WT and Mdfi-OE C2C12 cells, respectively. **(D)** ChIP assay identified the binding of Myod to the Camk2b promoter region. **(E)** qPCR detection of the mRNA level of the *Camk2b* gene in C2C12 cells after Myod overexpressing. **(F)** The dual-luciferase reporter system identified the promoter activity of Camk2b after Myod overexpressing. *p < 0.05; **p < 0.01; ***p < 0.001. The results are presented as Mean ± S.E.M. of three replicates for each group.

To further examine the regulation mechanism of Mdfi on the transformation of muscle fiber types, we performed additional experiments. The qPCR results showed that overexpression of Mdfi increased the mRNA level of *Tnni1*, *MyHC I*, *MyHC IIa*, and *Mb* but decreased the mRNA level of *MyHC IIb* (Figure 6A). Furthermore, we used qPCR to identify the mitochondrial DNA copy number, and the results showed that overexpression of Mdfi increased the mitochondrial DNA copy number in C2C12 cells (Figure 6B). We also detected the expression level of *Camk2b* and its downstream cellular energy metabolism and mitochondrial oxidative phosphorylation-related genes. The qPCR results showed that overexpression of Mdfi increased the mRNA level of *Camk2b*, PPARG coactivator 1 alpha (*Pgc1a*), pyruvate dehydrogenase kinase (*Pdk4*), citrate synthase (*Cs*), cytochrome c oxidase subunit 4 (*Cox4*), acyl-Coenzyme A dehydrogenase medium-chain (*Acadm*), acyl-Coenzyme A oxidase 1 (*Acox1*), cytochrome c (*Cycs*) and ATP synthase H^+^ transporting mitochondrial F1 complex alpha subunit 1 (*Atp5a1*) (Figure 6C). The Ch-IP results showed that Myod bound to the promoter region of *Camk2b* (Figure 6D). Overexpression of *Myod* increased the expression of *Camk2b* (Figure 6E). Dual-luciferase reporter system results showed that overexpressing *Myod* increased the promoter activity of *Camk2b* (Figure 6F). Combined with the above findings, Mdfi promoted the expression of *Myod*, thus upregulating the expression of *Camk2b*. Subsequently, *Camk2b* promoted the expression of downstream genes, such as *Pgc1a*, *Pdk4*, *Cs*, *Cox4*, *Acadm*, *Acox1*, *Cycs*, and *Atp5a1*. These results suggested that overexpression of Mdfi positively regulated the transformation of muscle fiber types from fast-twitch muscle to slow-twitch muscle.

## Discussion

In myoblast development, Mdfi blocked the myogenic differentiation of non-muscle-derived stem cells by inhibiting exogenous MRFs expression (Chen et al., 1996). However, Huang et al. (2019) found that MDFIC, which has the same domain as Mdfi, promoted the myogenic differentiation of muscle-derived stem cells. Its regulatory mechanism has not been studied in the muscle-derived stem cells. These opposite regulation functions of myogenic differentiation may be caused by the difference between species and cell types. Similarly, the function and regulatory mechanism of Mdfi on muscle fiber type transformation are also unclear. Therefore, we aimed to reveal the regulatory mechanism of Mdfi on myogenic cell differentiation and muscle fiber type transformation. In the present study, we successfully constructed a Mdfi-OE C2C12 cell line and found that overexpression of Mdfi promotes C2C12 cell differentiation and positively modulates fast-to-slow-twitch muscle fibers transformation.

### Mdfi-OE Promoted C2C12 Cells Myogenic Differentiation by Upregulating the Expression of Myod, Myogenin, and Myosin

**FIGURE 7:**
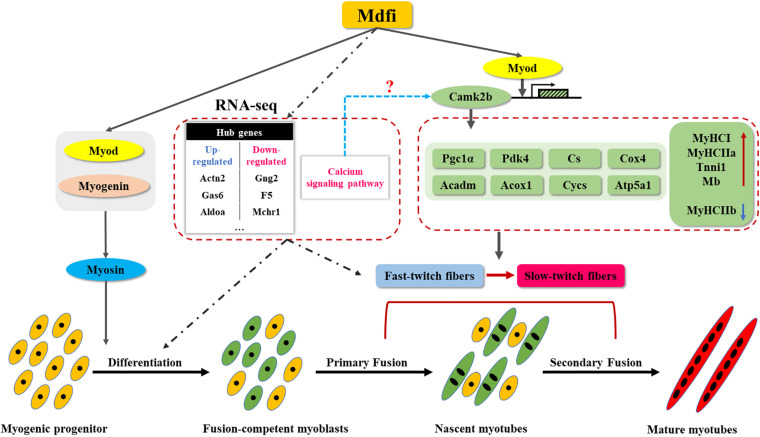
Mdfi overexpression promoted C2C12 cell differentiation and positively modulated fast-to-slow-twitch muscle fibers transformation. We constructed a Mdfi-overexpressing C2C12 cell line by the CRISPR/Cas9 system. During C2C12 myogenic development, Mdfi promotes the differentiation of C2C12 cells by upregulating the expression of Myod, Myog, and Myosin. Mdfi positively modulated fast-to-slow-twitch muscle fibers transformation by upregulating the mRNA levels of *Myod*, *Camk2b*, and its downstream genes, such as *Pgc1a*, *Pdk4*, *Cs*, *Cox4*, *Acadm*, *Acox1*, *Cycs*, and *Atp5a1*. In addition, the calcium signaling pathway was the most significant in C2C12 cells with Mdfi-OE. We also systematically established regulatory networks and identified hub genes in them. Their regulation mechanism in Mdfi regulation of C2C12 cell differentiation and the muscle fiber type transformation warrants further investigations.

As myoblasts differentiated and fused into myotubes, myosin began to express (Crow and Stockdale, 1986; D’Albis et al., 1989). Therefore, myosin was considered as a marker of muscle differentiation and maturation in the study of muscle development (Pizon et al., 2005; Zhao et al., 2014). In the present study, we selected myosin as a marker of myoblast differentiation and the immunofluorescent staining of myosin showed that overexpression of Mdfi significantly increased the percentage of myosin-positive C2C12 cells. In the process of myogenic differentiation, the dynamically temporal and spatial expression of MRFs family proteins control the determination and differentiation of myoblasts (Hernandez-Hernandez et al., 2017a). Previous studies have shown that Di-(2-ethylhexyl)-phthalate (DEHP) inhibited C2C12 cell differentiation by repressing the expression of Myod and Myog, resulting in decreased myotube formation and MyHC expression (Chen et al., 2013). In our study, the qPCR and Western blot results showed that Mdfi-OE significantly increases the expression of Myod, Myog, and Myosin in C2C12 cells, mainly in the middle and late stages of differentiation (D3 and D5). Meanwhile, the expression of Mdfi is dynamic in the process of myogenic differentiation. Therefore, we speculate that Mdfi plays an important role in the middle and late stage of C2C12 cell differentiation. Furthermore, the Co-IP results showed that Mdfi interacted with both Myod and Myog. Therefore, we concluded that Mdfi regulates the differentiation and maturation of C2C12 cells by dynamically regulating Myod, Myog, and Myosin (Figure 7).

### Pathway Enrichment and Gene Network Construction in RNA-seq Analysis

In our RNA-Seq analysis results, the “calcium signaling pathway” was the most enriched. The high intracellular calcium concentration induced myogenic differentiation and promoted the transformation of muscle fibers from fast-twitch to slow-twitch (Tu et al., 2016; Ravel-Chapuis et al., 2017). Numerous upregulated DEGs were enriched in the calcium signaling pathway, such as purinergic receptor P2X ligand-gated ion channel 5 (*P2rx5*), ryanodine receptor 3 (*Ryr3*), stromal interaction molecule 1 (*Stim1*), *Atp2a2*, and *Camk2b*, were involved in the process of myogenic differentiation. P2rx5, a ligand-gated ion channel, caused calcium to flow through the plasma membrane and promoted the terminal differentiation of myoblasts (Ryten et al., 2002). Ryr3 is a sarcoplasmic reticulum calcium release channel in skeletal muscle (Protasi et al., 2000). Stim1 is a sarcoplasmic reticulum membrane protein that can sense the change of calcium content. Overexpression of Stim1 promoted C2C12 cell differentiation and enhanced myotube formation, whereas *Stim1*-knockdown resulted in the opposite effects (Phuong et al., 2013). Atp2a2, also known as SERCA2, is a calcium transport ATPase, which functions as the reuptake of calcium from the cytoplasm to the sarcoplasmic reticulum (Harrer et al., 1995). Atp2a2 previously has been shown to express primarily in the slow-twitch skeletal muscle, and the knockdown of *Atp2a2* in C2C12 cells inhibited the expression of slow-twitch muscle marker genes (Wei et al., 2015). Camk2b, a calcium-dependent kinase, promoted mitochondrial biogenesis and involved in muscle fiber type transformation (Al-Shanti and Stewart, 2009). These genes were significantly upregulated in our RNA-Seq results and involved in regulating the formation of muscle fibers through calcium-transport. This result suggests that Mdfi-OE may promote C2C12 cell differentiation by regulating the calcium signaling pathway.

We also successfully established a PPI network and identified nine hub genes. Six upregulated hub genes are actinin alpha 2 (*Actn2*), growth arrest-specific 6 (*Gas6*), pregnancy-specific glycoprotein family members (*Psg17*, *Psg28*), collagen type VII alpha 1 (*Col7a1*), and aldolase A fructose-bisphosphate (*Aldoa*). Actn2, a muscle-specific actin-binding protein, bound explicitly disintegrin and metallopeptidase domain 12 (Adam12) to promote myoblast fusion (Galliano et al., 2000). Gas6 was increasingly secreted during C2C12 cell differentiation, and it has the same expression trend during the regeneration of muscle injury (Chikazawa et al., 2020). Psg17 and Psg28 are the members of the pregnancy-specific glycoprotein family. Psg17 bound CD9 protein to regulate the pregnancy process of the mouse (Wynne et al., 2006). To our knowledge, there are no published reports on Psg28. Col7a1, one of the extracellular matrix components, had different expression patterns in various types of muscle fibers (Zhu et al., 2016). Aldoa interacts with RYR to promote calcium release from the sarcoplasmic reticulum into the cytoplasm (Kramerova et al., 2008). Meanwhile, three downregulated hub genes are G protein subunit gamma 2 (*Gng2*), coagulation factor V (*F5*), and melanin-concentrating hormone receptor 1 (*Mchr1*). Gng2 is a member of guanine nucleotide-binding protein (G protein), which plays an essential role in various transmembrane signaling systems, mainly by binding G Protein-Coupled Receptors (GPCR; Liang et al., 2016). F5 is a critical factor in the blood coagulation process, primarily promoting the conversion of prothrombin to thrombin (Rodgers et al., 1987). Mchr1 is a GPCR mainly expressed in the brain and also expressed in muscle tissue (Saito et al., 2013). Some of these hub genes are involved in muscle formation and regeneration of muscle injury, while others were novel genes not involved in muscle development. All the above hub genes are significantly changed in our RNA-Seq results. The role of these genes in the process of Mdfi regulating C2C12 cell differentiation warrants further investigations.

### Mdfi-OE Positively Modulates Fast-to-Slow-Twitch Muscle Fibers Transformation

Skeletal muscle is a dynamically changing system with high plasticity. Skeletal muscle responds to external environmental stimuli, nutrient levels, mechanical training, and age, and adjusts the content of different MyHC subtypes in muscle fibers. In addition, the transformation of muscle fiber types occurs due to the changes in intracellular signal pathways caused by internal physiological changes, pathological stimulation, and stress (Grange et al., 2001; Pette, 2002). In the present study, we performed immunofluorescent staining to examine different subtypes of MyHC and found that overexpression of Mdfi increased the percentage of MyHC I and MyHC IIa positive C2C12 cells. In contrast, overexpression of Mdfi decreased the percentage of MyHC IIb positive C2C12 cells.

As expected, in the RNA-seq results, we found that *Myh4*, *Myh2*, and *Mb* expressed differently between WT and Mdfi-OE C2C12 cells. Moreover, the qPCR results showed that Mdfi overexpression increased the expression of MyHC I (*Myh7*), MyHC IIa (*Myh2*), *Tnni 1*, and *Mb*, while decreased the expression of MyHC IIb (*Myh4*). These qPCR results were consistent with the results of the RNA-seq data. This evidence indicated that the overexpression of Mdfi promotes the transformation of muscle fibers from type IIb to type IIa and type I. Furthermore, we explored the regulatory mechanism of Mdfi in regulating the transformation of muscle fiber types. In the qPCR results, we found that *Camk2b* was upregulated, enriched in three pathways related to the transformation of muscle fiber types, and identified as the hub genes in the network. The gene *Camk2b*, a downstream dependent kinase of the calcium signaling pathway, promoted mitochondrial biogenesis and participated in the transformation of muscle fiber types from fast-twitch muscle to slow-twitch muscle (Al-Shanti and Stewart, 2009). In mouse muscle development, *Camk2* regulated the oxidative metabolism of mouse muscle mediating by AMPK signaling (Raney and Turcotte, 2008). Therefore, we examined the expression of *Camk2b*, and the qPCR results demonstrated that overexpression of Mdfi significantly increased its expression.

Furthermore, we detected the expression level of downstream cellular energy metabolism and mitochondrial oxidative phosphorylation-related genes of *Camk2b*, such as *Pgc1a*, *Pdk4*, *Cs*, *Cox4*, *Acadm*, *Acox1*, *Cycs*, and *Atp5a1*. The knockin of *Pgc1a* in mice increased the distribution of red oxidized fibers and promoted the transformation of muscle fibers from type IIx to type I. Conversely, the knockout of *Pgc1a* in skeletal muscle induced the transformation of muscle fibers from type I and IIa to type IIx and IIb (Arany et al., 2007). *Pdk4* was activated by the co-expression of *Pgc1a* and estrogen-related receptor alpha (*Erra*) and played an important role in glucose oxidative phosphorylation (Sugden and Holness, 2003). In rat liver mitochondria, the inhibition of *Cox2* induced mitochondrial toxicity by inhibiting oxidative phosphorylation (Syed et al., 2016). During the development of porcine skeletal muscle, the addition of niacin supplementation promoted genes involved in mitochondrial fatty acid catabolism, citric acid cycle, and oxidative phosphorylation, such as *Cact*, *Sdha*, *Cox4*, and *Cox6a1*. Their expression induced the transformation of muscle fibers from type II to type I (Khan et al., 2013). The genes *Acadm*, *Acox1*, *Cycs*, and *Atp5a1* were marker genes of mitochondrial fatty acid oxidation and oxidative phosphorylation in the muscle (Kitamura et al., 2020). The qPCR results showed an increase in expression of these genes. In addition, the Ch-IP results demonstrated that Myod binds to the promoter of *Camk2b* to regulate the transcriptional activation of *Camk2b*.

All the above results indicated that Mdfi promotes the transcriptional activation of *Camk2b* by binding to Myod, increasing the expression of downstream *Pgc1a*, *Pdk4*, *Cs*, *Cox4*, *Acadm*, *Acox1*, *Cycs*, and *Atp5a1*, and ultimately promoting the transformation of muscle fibers from type IIb to type IIa and type I (Figure 7). In addition, whether Mdfi activates *Camk2b* through the calcium signaling pathway to regulate muscle fiber type transformation needs to be further explored.

## Conclusion

In conclusion, our findings have further characterized the regulatory function of Mdfi in C2C12 cell differentiation and muscle fiber type transformation. Mdfi-OE promoted the differentiation of C2C12 cells by upregulating the expression of Myod, Myog, and Myosin. Meanwhile, Mdfi-OE promoted the expression of *Camk2b* by binding to Myod. *Camk2b*, in turn, upregulated the expression of downstream genes, such as *Pgc1a*, *Pdk4*, *Cs*, *Cox4*, *Acadm*, *Acox1*, *Cycs*, and *Atp5a1*, and ultimately promoting the transformation of muscle fibers from the fast-twitch to the slow-twitch. In addition, combined with RNA-seq results, we found that the calcium signaling pathway was the most significant in C2C12 cells with Mdfi-OE. Its regulation mechanism in Mdfi regulation of C2C12 cell differentiation and the muscle fiber type transformation warrants further investigations. We also systematically established the regulatory networks of Mdfi-OE on C2C12 cell differentiation and muscle fiber type transformation and identified hub genes. These results led us to propose a regulatory mechanism model of how Mdfi regulates muscle development. Mdfi may be a therapeutic target for muscle- and metabolic-related diseases treatment.

## Data Availability Statement

The datasets presented in this study can be found in online repositories. The names of the repository/repositories and accession number(s) can be found below: http://www.ncbi.nlm.nih.gov/bioproject/679162.

## Author Contributions

CW, BH, and YJ designed the research. YJ and YZ performed the research and developed the methods. BH, ZN, and ZY analyzed the data. BH, QL, CH, and CW wrote the manuscript. All authors made contributions to this study and read and approved the final manuscript.

## Conflict of Interest

The authors declare that the research was conducted in the absence of any commercial or financial relationships that could be construed as a potential conflict of interest.

## Supplementary Material

Supplementary Figure 1 Cell fusion observation under white light.

Supplementary Table 1 All the primers used in this study.

Supplementary Table 2 Antibodies used in the study.

Supplementary Table 3 The DEGs in Mdfi-OE vs. WT.

Supplementary Table 4 The KEGG pathways were enriched by DEGs.
