# Supplementary figures and images for: Mdfi Promotes C2C12 Cell Differentiation and Positively Modulates Fast-to-Slow-Twitch Muscle Fiber Transformation
Source: Front Cell Dev Biol. 2021 Jan 22;9:605875. doi: 10.3389/fcell.2021.605875 (PMC7862576; doi:10.3389/fcell.2021.605875)

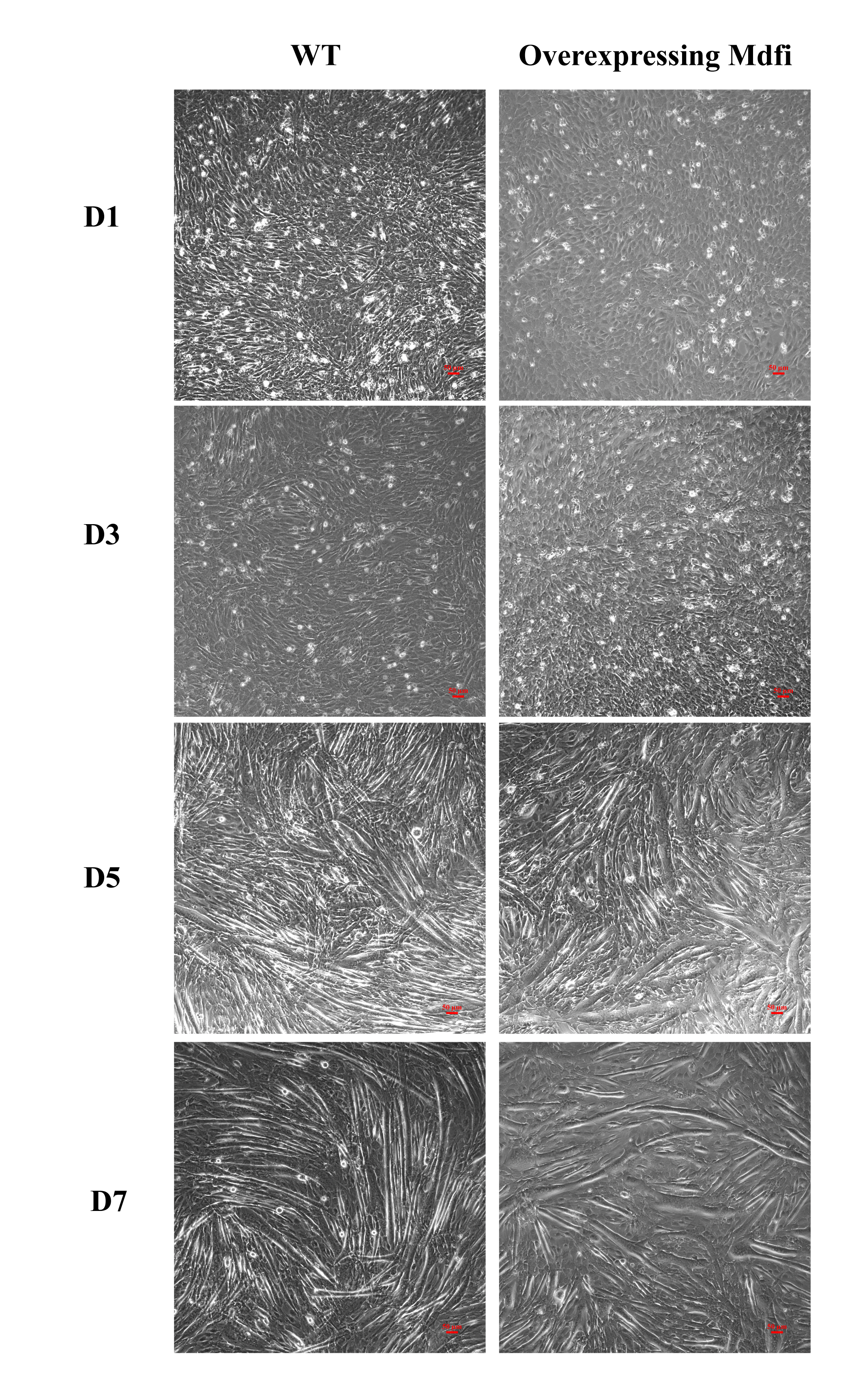

Supplement: Supplementary Figure 1 — Cell fusion observation under white light. [file Image_1.TIF]
