# Supplementary material for: Mdfi Promotes C2C12 Cell Differentiation and Positively Modulates Fast-to-Slow-Twitch Muscle Fiber Transformation
Source: Front Cell Dev Biol. 2021 Jan 22;9:605875. doi: 10.3389/fcell.2021.605875 (PMC7862576; doi:10.3389/fcell.2021.605875)
Supplement: Supplementary Table 1 — All the primers used in this study. [file Table_1.DOCX]

Table S1.

All the primers used in this study.

| Gene name | | Forward primer sequerence (5'-3') | Reversed primer sequerence (5'-3') | |
| --- | --- | --- | --- | --- |
| β-Actin | | TGCTGTCCCTGTATGCCTCTG | GCTGTAGCCACGCTCGGTC | |
| Mdfi | | CCACGACCACCTCTCAGAAC | GACAGGACAGTATGCAGTGGA | |
| Myod | | GGCTACGACACCGCCTACTAC | GGTCTGGGTTCCCTGTTCTG | |
| Myogenin | | CCTGGAAGAAAAGGGACTGG | CGCTCAATGTACTGGATGGC | |
| Myf5 | | AAGGCTCCTGTATCCCCTCAC | TGACCTTCTTCAGGCGTCTAC | |
| Myf6 | | AGAGGGCTCTCCTTTGTATCC | CTGCTTTCCGACGATCTGTGG | |
| Myosin | | AAGTCGGCTGAAATCGCAAAG | CGGATTCTGAGATTATGCAGCAC | |
| MyhcI | | GAATGGCAAGACGGTGACTGTG | GGAAGCGTAGCGCTCCTTGAG | |
| MyhcIIa | | ATCAACCAGCAGCTGGACACCA | TCCAGCACGAACATGTGGTGGT | |
| MyhcIIb | | ACAGACTAAAGTGAAAGCCTACAA | CACATTTTGTGATTTCTCCTGTCAC | |
| MyhcIIx | | CCAATGAAACCAAGACTCCTGG | TGCTATCGATGAACTGTCCCTC | |
| Tnni1 | | CTTCAGGACTTGTGCCGAGAG | GCTTGAACTTCCCACGGAG | |
| Tnni2 | | GAAGATCGACGTGGCTGAAGAG | ACTTGCCCCTCAGGTCAAATAG | |
| PGC-1α | | CGCAGGTCGAATGAAACTGACTT | GTTACCTGCGCAAGCTTCTCTGA | |
| Pdk4 | | AGGATTACTGACCGCCTCTTTAG | ATTCCGGGAATTGTCCATCACAG | |
| Cs | | AACTCAGGACGGGTTGTTCCAG | TAGTAATTCATCTCCGTCATGCC | |
| Cox2 | | ATCCCAGGCCGACTAAATCAAG | AGAGCATTGGCCATAGAATAAC | |
| Cox4 | | CTATGTGTATGGCCCCATCC | CAGCGGGCTCTCACTTCTTC | |
| Camk2b | | GCACGTCATTGGCGAGGAT | ACGGGTCTCTTCGGACTGG | |
| Myod-Camk2b | | CCTGGCAAGTCCCTGTA | TTGGGTTTGGTTTGTTTT | |
| β-actin | GGCTGTATTCCCCTCCATCG | | | CCAGTTGGTAACAATGCCATGT |
| Slc6a19 | CAGGTGCTCAGGTCTTCTACT | | | CGATCACAGAATCCATCTCACAA |
| Grin2c | GGGATCTGCCATAACGAGAAG | | | GCACTGAGTGTCGAAGTTTCCA |
| Pla2g4e | ATGGTGACAGACTCCTTCGAG | | | CCTCTGCGTAAAGCTGTGG |
| Gnai1 | GGTTTACAGACACGTCCATCAT | | | GCCTGCATATTCTGGGTAGCAT |
| Dnase1l3 | CTCTGCTCCTTCAATGTGAGG | | | GCTGCTGTCCTTGATTTCCAT |
| Nrsn1 | CTATGGAGTTCGGTCCTACCTG | | | CCTGTTAGGCGATCTCTGGAT |
| Cyp11a1 | AGGTCCTTCAATGAGATCCCTT | | | TCCCTGTAAATGGGGCCATAC |
| Ceacam16 | CTTAACAGGCAGTTTCAGACGG | | | GAGACGAAGGGTATCCCTACG |
| Adamtsl2 | CTTCAACTCCCGTGTGTATGAC | | | GCAAACCCCTCTCAGGTCG |
| Ctrb1 | ATGGCATTCCTTTGGCTTGTG | | | GGATAGCATCCTCTCCGTTGAC |
| Cox6a2 | CTGCTCCCTTAACTGCTGGAT | | | GATTGTGGAAAAGCGTGTGGT |
| Ctsc | CAACTGCACCTACCCTGATCT | | | TAAAATGCCCGGAATTGCCCA |
| Arap2 | CTGAGTGAAAAGCACGATCCT | | | TCTTCAGTGTCTTCGGACCTT |
| Arx | GGCCGGAGTGCAAGAGTAAAT | | | TGCATGGCTTTTTCCTGGTCA |
| Wnt5a | CAACTGGCAGGACTTTCTCAA | | | CATCTCCGATGCCGGAACT |

The PCR reaction system of Luciferase Reporter Assay

| Primer Name | Primer sequence (5’-3’) |
| --- | --- |
| Camk2b-F | CGGGGTACCCGCCGAGGCTCTTACTT |
| Camk2b-R | CCCAAGCTTGCCTCTAATGCTTCGGTC |
| Myod-F | CCGCTCGAGGGACACGACTGCTTTCTTC |
| Myod-R | CGCGGATCCAAGCACCTGATAAATCGC |

The primary antibodies used in this study.

| Primary antibody | Type | Company | Catalog No. | Dilution rate |
| --- | --- | --- | --- | --- |
| β-actin | Monoclonal | Bioss | bsm-33036M | 1:2000 |
| Myod | Polyclonal | SANTA CRUZ | #C2613 | 1:200 |
| Myogenin | Polyclonal | SANTA CRUZ | #J2314 | 1:200 |
| Myosin | Monoclonal | BOSTER | BM0096 | 1:500 |
| Myhc I | Monoclonal | abcam | ab11083 | 1:5000 |
| Myhc IIa | Polyclonal | abcam | ab124937 | 1:5000 |

The secondary antibodies used in this study.

| Secondary Antibody | Type | Company | Catalog No. | Dilution rate |
| --- | --- | --- | --- | --- |
| Goat Anti-mouse IgG | HRP | Bioss | bs-0296G | 1:3000 |
| Goat Anti-rabbit IgG | HRP | Bioss | bs-0295G | 1:3000 |
