# Supplementary material for: Mdfi Promotes C2C12 Cell Differentiation and Positively Modulates Fast-to-Slow-Twitch Muscle Fiber Transformation
Source: Front Cell Dev Biol. 2021 Jan 22;9:605875. doi: 10.3389/fcell.2021.605875 (PMC7862576; doi:10.3389/fcell.2021.605875)
Supplement: Supplementary Table 2 — Antibodies used in the study. [file Table_2.DOCX]

Table S2.

Primary antibodies used in the study.

| Primary antibody | Type | Company | Catalog No. | Dilution rate |
| --- | --- | --- | --- | --- |
| Ccnb1 | Polyclonal | CST | #4138 | 1:1000 |
| Ccnd1 | Polyclonal | CST | #2922 | 1:1000 |
| P21 | Polyclonal | Bioss | bs-0741R | 1:500 |
| β-actin | Monoclonal | Bioss | bsm-33036M | 1:2000 |
| Myod | Polyclonal | SANTA CRUZ | #C2613 | 1:200 |
| Myogenin | Polyclonal | SANTA CRUZ | #J2314 | 1:200 |
| Myosin | Monoclonal | BOSTER | BM0096 | 1:500 |
| FAK | Polyclonal | CST | #3285 | 1:1000 |
| Phospho- FAK(Tyr397) | Polyclonal | CST | #8556 | 1:1000 |
| Paxillin | Polyclonal | CST | #2542 | 1:1000 |
| Phospho- Paxillin (Tyr397) | Polyclonal | CST | #8556 | 1:1000 |
| Myhc I | Monoclonal | abcam | ab11083 | 1:5000 |
| Myhc IIa | Polyclonal | abcam | ab124937 | 1:5000 |

Secondary antibody used in the study

| Secondary Antibody | Conjugate Used | Company | Catalog No. | Dilution rate |
| --- | --- | --- | --- | --- |
| Goat Anti-mouse IgG | HRP | Bioss | bs-0296G | 1:3000 |
| Goat Anti-rabbit IgG | HRP | Bioss | bs-0295G | 1:3000 |
| Goat Anti-Mouse IgG/Cy3 | Cy3 | Bioss | bs-0296G-Cy3 | 1:200 |
| Goat Anti-Rabbit IgG/Alexa Fluor 594 | Alexa Fluor 594 | Bioss | bs-0295G-AF594 | 1:200 |
| Goat Anti-Mouse IgG/FITC | FITC | Bioss | bs-0296G-FITC | 1:200 |
